# Efficacy and Safety of Pulsed Radiofrequency of Dorsal Root Ganglion in Elderly Patient Population With Acute and Subacute Zoster-Related Pain

**DOI:** 10.1155/2024/6586167

**Published:** 2024-09-06

**Authors:** Gözde Dağıstan, Serdar Erdine

**Affiliations:** ^1^ Anesthesiology and Reanimation Department (Algology) Akdeniz University Faculty of Medicine, Antalya, Turkey; ^2^ Istanbul Pain Center Anesthesiology and Reanimation Department (Algology), Istanbul, Turkey

**Keywords:** dorsal root ganglion, herpes zoster, neuropathic pain, pulsed radiofrequency

## Abstract

**Background:** Herpes zoster (HZ) is typically characterized by a burning, stabbing pain, hyperalgesia, and allodynia. In some patients, despite the lesions resolving, the pain persists and becomes chronic. If the pain continues for more than 6 months after the onset of the pain phase, this condition is called postherpetic neuralgia (PHN). The frequency and severity of PHN increase with advancing age. The pain in PHN can be severe, sometimes resistant to medications, significantly impacting the patients' quality of life. The elderly patient population cannot tolerate the medications due to their side effects. In this situation, interventional pain treatment should be applied in the elderly patient group who have a high risk of developing PHN compared to other age groups.

**Method:** We included patients over 65 years of age with HZ-related pain who underwent dorsal root ganglion (DRG) pulsed radiofrequency (PRF) within the first 6 months from the onset of pain. We divided these patients into 2 groups: patients who underwent intervention within the first 1 month from the onset of pain and patients who underwent intervention between 1 and 6 months. We recorded medication doses and Numeric Rating Scale (NRS) scores before the procedure and at 1 week, 1 month, 3 months, and 6 months after the procedure.

**Results:** After the DRG PRF treatment, NRS scores improved significantly in both groups (*p* < 0.05). The mean NRS score in the early DRG PRF group was significantly lower than that in the late DRG PRF group (*p* < 0.05). The medication doses in the early DRG PRF group were significantly lower than those in the other group (*p* < 0.05).

**Conclusions**: Interventional pain treatment should be applied as soon as possible in the elderly patient group who do not respond to first-line medical treatment or cannot tolerate medical treatment due to its side effects and who have a high risk of developing PHN compared to other age groups. DRG PRF, applied in the early period of medical treatment-resistant acute HZ, is safe and effective, preventing the progression to PHN.

## 1. Introduction

Varicella zoster virus (VZV) induces primary infection upon encountering the organism. In herpes zoster (HZ), the virus, which remains latent in the sensory ganglia following primary infection, causes recurrent infection in the organism with age and decreased immunity [1]. When VZV is activated, its virions, through transport along sensory nerves, travel to neuronal cell bodies in the skin, creating a typical dermatomal rash. Necrosis, inflammation, and demyelination are observed in the peripheral sensory nerves [1, 2]. In HZ, the inflammation caused by VZV sensitizes the nociceptors in the skin. Peripheral sensitization reduces the heat activation threshold in C-nociceptors. If this threshold falls below the skin temperature, spontaneous firing occurs in afferents. Deafferentation occurs with the destruction of sensory neurons in the VZV-infected dorsal root ganglion (DRG) [3]. All these mechanisms lead to spontaneous burning pain and allodynia in the dermatomal area affected by the rash.

HZ is typically characterized by unilateral, dermatomal spread, accompanied by a burning, stabbing pain, hyperalgesia, and allodynia. In some patients, despite the lesions resolving, the pain persists and becomes chronic. If the pain continues for more than 6 months after the onset of pain, this condition is referred to as postherpetic neuralgia (PHN) [4]. PHN is the most common complication of HZ [5]. The pain in PHN can be severe, sometimes resistant to medications, significantly impacting the patients' quality of life [1]. The frequency and severity of PHN increase with advancing age. It has been reported that 30% of patients progressing from HZ to PHN are aged 80 and above [6]. In addition to age, the severity of acute pain and rushes are risk factors for the development of PHN [7]. Various treatments are employed for both PHN and HZ, including epidural injections, nerve blocks, pulsed radiofrequency (PRF) applied to the DRG, and spinal cord stimulation (SCS) [8]. Numerous studies demonstrate the effectiveness of DRG PRF in both HZ-related pain and PHN-related pain [1, 4, 10, 11]. Different applications in terms of duration are reported, including during the acute and subacute phases of HZ or in patients developing PHN [1, 4, 9–11].

PRF is believed to achieve long-term pain suppression by suppressing pain signals from the peripheral nervous system to the central nervous system. Inhibiting nociceptive C and A-delta fibers with PRF has been shown to contribute to the reduction of pain [12].

## 2. Materials and Methods

### 2.1. Patients

The study was approved by the Research Ethics Committee at Akdeniz University with number TBAEK-356.

Medical records of patients who underwent DRG PRF within the first 6 months after the onset of pain due to HZ were examined between January 2021 and April 2023 in the university pain clinic and special pain clinic. Inclusion criteria for the study were patients who were 65 years or older, had a Numeric Rating Scale (NRS) ≥ 4, had pain resistant to conventional treatments, and had undergone DRG PRF within the first 6 months after the onset of HZ-related pain. Exclusion criteria included patients with trigeminal HZ, incomplete medical records, cancer-causing immunodeficiency, organ transplantation, or systemic diseases. Additionally, patients who had undergone interventions other than DRG PRF, such as epidural steroid injections, sympathetic ganglion block, or SCS, within 6 months were excluded.

Patients were divided into two groups: those who underwent DRG PRF within the first 1 month of pain onset as Group 1 and those who underwent DRG PRF between 1 and 6 months after pain onset as Group 2. Group 1 consisted of patients who could not tolerate medical treatment due to its side effects or were unable to reach an effective drug dose due to side effects. A total of 40 patients were included in the study, with 19 receiving DRG PRF within the first month and 21 receiving it between 1 and 6 months. Patient information before and after the procedure was obtained from medical records, including age, gender, affected dermatome region (cervical/thoracic/lumbar), and the duration from the onset of HZ-related pain. The equivalent pregabalin dose for all gabapentin and pregabalin derivative drugs used by the patients and the morphine equivalent dose for narcotic analgesics were calculated [13–15]. Medication doses and NRS scores were recorded before the procedure and at 1 week, 1 month, 3 months, and 6 months after the procedure.

### 2.2. Statistical Analysis

Statistical analysis was conducted using SPSS software. An independent sample *t*-test was used to assess age, sex, HZ distribution area, and time from pain onset. Differences in pain intensity and medication doses between groups were assessed with an independent sample *t*-test. Changes in pain intensity and medication doses within each group were evaluated using paired sample *t*-tests. The values were expressed as mean and standard deviation (mean ± SD), and a significance level of *p* < 0.05 was considered statistically significant.

### 2.3. Procedure

**Figure 1:**
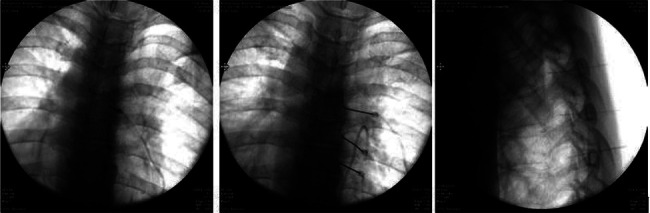
Pulsed radiofrequency of thoracic dorsal root ganglion. Oblique, anteroposterior, and lateral fluoroscopic views of the electrodes.

**Figure 2:**
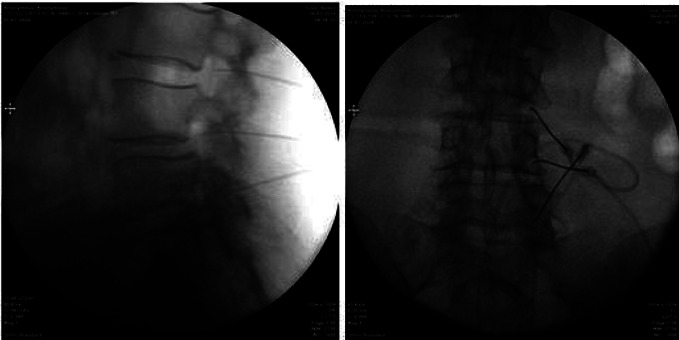
Pulsed radiofrequency of lumbar dorsal root ganglion. Anteroposterior and lateral fluoroscopic views of the electrodes.

Patients undergoing cervical DRG PRF were positioned supine, while those undergoing thoracic and lumbar DRG PRF were positioned prone on the procedure table. Hemodynamic parameters (blood pressure, saturation, and heart rate) were monitored. Intravenous cannulation was performed. The dermatome with scars from HZ lesions was identified for the intervention. For all procedures, PRF was applied to the adjacent DRGs, one level above and one level below the predetermined level of the DRG. The affected level was determined by observing scar tissue and examining the patient. Additionally, during the procedure, the level was confirmed by giving sensory stimulation to the patient. All procedures were performed under fluoroscopic guidance. In the thoracolumbar region, the lower endplate of the target vertebra for the procedure was aligned. Subsequently, with the fluoroscope in an oblique position, after local anesthetic injection, a 22-gauge, 10-cm electrode with a 10-mm active tip radiofrequency needle was introduced under the pedicle. Under lateral imaging, the needle tip was advanced up to half of the foraminal width (Figures 1 and 2).

**Figure 3:**
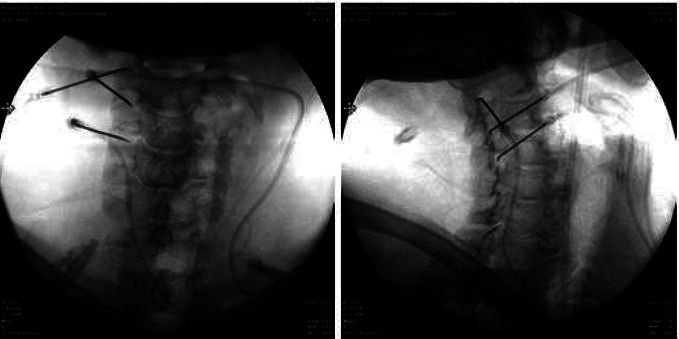
Pulsed radiofrequency of cervical dorsal root ganglion. Anteroposterior and oblique fluoroscopic views of the electrodes.

To confirm the proximity of the needle tip to the DRG, sensory stimulation at a frequency of 50 Hz was applied, and the patient was queried whether they felt the stimulus in the dermatome where the needle was placed, keeping the stimulus below 0.5 V. Motor stimulation at a frequency of 2 Hz was applied to check for muscle fibrillation. No muscle fibrillation was observed. For cervical DRG PRF, the maximum width of the intervertebral foramen at the targeted level was determined by adjusting the fluoroscopic position. Following subcutaneous local anesthetic injection, a 22-gauge, 10-cm electrode with a 10-mm active tip radiofrequency needle was introduced posterior to the foramen. The needle was positioned so as not to cross the midline of the facet column (Figure 3).

Once again, sensory and motor stimulation were applied to confirm the needle location. The parameters of PRF were set as follows: temperature, 42°C; pulse width, 20 milliseconds; frequency, 2 Hz; voltage, 45 V; and duration, 360 s.

## 3. Results

The ages, genders, initial mean pain scores, and the distribution area of HZ were similar in both groups (*p* > 0.05) (Table 1). Statistically significant reductions in postprocedural pain were observed in both groups (Table 2).

**Figure 4:**
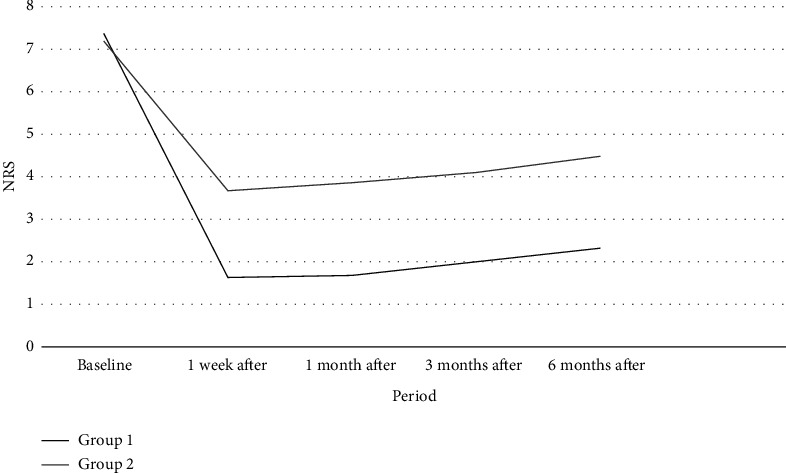
NRS-11 score pre- and postoperation (Group 1: patients who underwent intervention within the first 1 month from the onset of pain; Group 2: patients who underwent intervention between 1 and 6 months from the onset of pain).

Despite the similar initial pain scores between the two groups, there was a significant difference in all NRS scores in the postprocedural follow-ups (*p* < 0.05). In Group 1, patients who underwent DRG PRF showed significantly lower pain scores in postprocedural follow-ups within the first month after the onset of pain (Figure 4).

**Figure 5:**
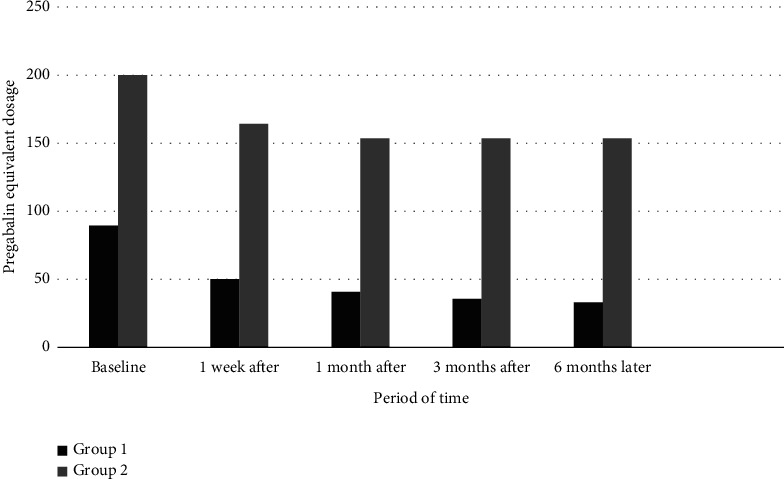
Patients' use of pregabalin equivalent dosages pre- and postoperation (Group 1: patients who underwent intervention within the first 1 month from the onset of pain; Group 2: patients who underwent intervention between 1 and 6 months from the onset of pain).

**Figure 6:**
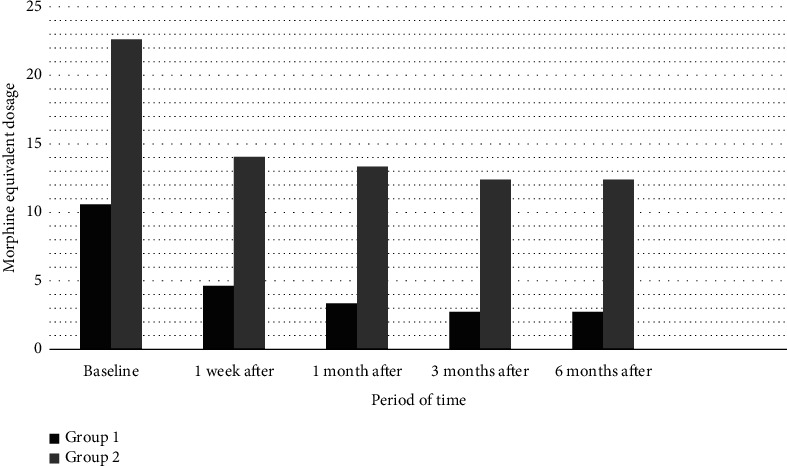
Patients' use of morphine equivalent dosages pre- and postoperation (Group 1: patients who underwent intervention within the first 1 month from the onset of pain; Group 2: patients who underwent intervention between 1 and 6 months from the onset of pain).

When comparing the medication doses used by patients, in both groups, doses in the postprocedural follow-ups significantly reduced compared to preprocedural doses (*p* < 0.05) (Figures 5 and 6).

Group 1's pregabalin and morphine preprocedural equivalent doses were significantly lower than those of Group 2. A significant difference was observed between the two groups in preprocedural and postprocedural follow-ups.

No serious complications such as pneumothorax, bleeding, or infection were observed during the procedure.

## 4. Discussion

Considering the increasing frequency and severity of PHN with age, the importance of early intervention in the acute phase becomes evident in these patients. In a study conducted in Japan, the conversion rate of HZ to PHN was reported to be 15.7% and 13.6% in the age groups of 50–59 and 60–69, respectively, while it increased to 20.2% in the 70–79 age group and 32.9% in those aged 80 and above [16]. Another study reported that over 50% of patients aged 60 and above with HZ developed PHN, and some studies reported an even higher rate of 60%–75% [9, 10]. The development of PHN in patients under the age of 40 is very rare [17]. Therefore, more attention should be paid to pain management in the elderly patient group to prevent the chronicity of pain related to HZ.

Steroids can play an important role in managing inflammation and relieving pain during the acute phase of HZ. Some researchers have recommended using corticosteroids to relieve the zoster-associated pain in the acute phase of the disease. However, further research studies are needed to evaluate the efficacy of corticosteroids on the transition from acute pain to PHN [18].

Studies have shown that interventional procedures performed in the acute phase reduce the conversion rate of HZ to PHN and provide more effective pain palliation [4, 11, 19].

However, there is no consensus on the time interval of the acute phase in these studies. While some studies consider the first month from the onset of pain as the acute phase [20], others define it as 1–3 months [10] and some as 1–6 months [21].

Similarly, studies vary in defining PHN as occurring more than 1 month [1, 21], more than 3 months [10, 11], or more than 6 months after the onset of pain [4]. If adequate pain palliation is not achieved during the acute phase of HZ, a neuropathic process such as central sensitization due to continuous nociceptive signals from damaged neurons may begin [21]. This is one of the main reasons for the development of PHN. In this retrospective study, we focused on patients aged 65 and above who had passed different time periods since the onset of HZ-related pain, posing a much higher risk for the development of PHN.

In a study conducted in 2023 comparing subacute HZ and PHN groups, both treated with DRG high-voltage long-duration PRF, DRG PRF was found to be effective in both groups [10]. However, better results were obtained in the subacute HZ group. The authors defined the subacute HZ group as patients within a 1- to 3-month period from the onset of HZ and the PHN group as patients with 3 months or more since the onset. As a result, they suggested that early effective interventional treatments reduce the severity and duration of HZ. When comparing the results of standard PRF parameters applied to the acute HZ group (less than 90 days from the onset of HZ) and PHN group (more than 90 days from the onset), successful results were also obtained in the acute HZ group [11]. In the literature, two studies focusing on the group of patients who underwent the procedure within the first month of the onset of HZ were found. In the first study, patients with pain due to acute HZ were divided into two groups, one receiving DRG PRF and the other receiving DRG PRF plus paravertebral IL-2b injection [22]. DRG intervention was found to be effective in this study. The study, which did not make a comparison in terms of duration, reported that DRG PRF was effective in the acute period, but the results of the group with IL-2b added were superior. In another study, one group with pain due to acute HZ received high-voltage DRG PRF, and the other group received high-voltage DRG PRF plus ozone injection [20]. While no significant difference was observed between the two groups in Week 1 and Month 1 results, the group with added ozone showed more reduction in NRS in the third month results. These studies investigated the effectiveness of DRG PRF treatment alone and treatments added to DRG PRF, rather than comparing them in terms of duration. In our study, we focused on the population aged 65 and above, for whom the risk of PHN is much higher, who received the DRG PRF within the first month of the onset of pain and between 1 and 6 months after the onset of pain. We aimed to determine the optimal intervention time for this group.

Drugs play a crucial role in the treatment of neuropathic pain. However, their use in the geriatric patient population can be limited due to additional comorbidities, polypharmacy, and decreased liver and kidney functions. A systematic review classified the side effects related to gabapentinoids into those affecting the central nervous system and the gastrointestinal system. Central nervous system side effects include drowsiness, dizziness, somnolence, impaired balance, difficulty walking, impaired vision, and respiratory depression, while gastrointestinal side effects include constipation and nausea. The frequency of these side effects generally increases with dose and age. Therefore, the elderly patient group is more susceptible to these side effects compared to the younger patient group. Even in the elderly population with normal laboratory values, an increased risk of gabapentinoid toxicity due to reduced renal clearance has been reported [23]. Statistical data in our study showed a significant difference in both morphine and pregabalin equivalent doses between the two patient groups. The drug doses applied in the early DRG PRF group were significantly lower than those in the other group. The reason for this is that the population we performed the procedure on within the first month had either not used drugs like pregabalin or gabapentin at all or used low doses due to side effects, and dose escalation could not be performed due to side effects, resulting in inadequate pain palliation.

The effect of PRF on neuropathic pain is still not fully understood. PRF is assumed to create changes at the neuronal level by generating heat and a strong electromagnetic field at the tip of the electrode without causing neurodestruction. PRF exhibits a kind of neuromodulation effect in the nerve or DRG where it is applied [21, 24]. In a study comparing patients aged 50 and above with HZ-related pain in the acute phase who underwent DRG PRF and patients who underwent SCS, similar results were found, indicating that both techniques provided pain reduction [4]. The advantage of DRG PRF over SCS is its lower cost and lower risk of infection. In this study, the optimal treatment interval for HZ was suggested to be 30–180 days. However, all patients in this study were within the same time interval, and any two time intervals were not compared. We compared two time intervals. Patients who underwent DRG PRF showed significantly lower pain scores in postprocedural follow-ups within the first month after the onset of pain. However, in our study, the reduction in pain in patients who underwent DRG PRF within the same time interval for HZ-related pain was lower than that in patients from the previous study. This difference could be attributed to both the duration of the procedure and the age range of the patient population. The average age in this study was 65, and the duration of the procedure was an average of 69 days from the onset of pain, while the average age of our patients was 72.8, and the duration of the procedure was an average of 110 days.

There is no consensus on the duration of PRF application. Studies exist that apply PRF for 120 s [1, 25], 180 s [9], and 360 s [4, 11, 21, 22], and their effectiveness has been proven. The authors applied PRF for 120 s.

The potential mechanism considered in PRF involves the long-term suppression of pain signals from the periphery to the central nervous system by inhibiting nociceptive C fibers through PRF. It has been reported that PRF induces changes at the molecular level and alters neuronal activity. PRF has been shown to reduce the activation of activated microglia, which leads to neuroinflammation in the dorsal horn and initiates the process of chronic neuropathic pain. In neuropathic pain models, it has been reported that PRF reduces the increased proinflammatory gene expression in the DRG and improves mechanical allodynia and thermal hyperalgesia. It has been shown to increase endogenous opioid precursor mRNA and opioid peptides. PRF is reported to increase noradrenergic and serotonergic descending pain inhibitory pathways [12]. Although PRF is demonstrated not to cause damage to tissues, microscopic destructions in C fibers and A-delta fibers have been shown under an electron microscope [26]. It is believed that the microscopic damage in these fibers also plays a role in reducing pain.

Reactivation of the latent virus in the DRG is involved in HZ. Even if the pathology is at a single-level DRG, there is evidence that it affects other levels of DRGs. Anatomical and electrophysiological studies have shown that radicular pain does not only occur in spinal segments where pathology exists but also spreads to adjacent spinal segments [27]. This situation indicates that the pathology affects multiple levels of DRGs, even if it is in a single-level DRG. Therefore, interventions should be applied to adjacent DRGs, not just the affected DRG. In our study, we applied interventions to both the affected DRG and the adjacent DRGs at the upper and lower levels.

Although peripheral nerves and cutaneous nociceptors participate in the process of HZ, considering that the main center of pathology in HZ is predominantly the DRG, it can be said that the target structure to be treated here is the DRG. Procedures performed on the DRG and other target tissues in HZ have been compared. In a study comparing the application of PRF to the intercostal nerve and DRG in patients over 60 with HZ-related pain, both interventions were found to be effective, but in the DRG PRF group, VAS scores decreased more significantly compared to the intercostal PRF group, and quality of life scores increased more significantly [1]. The authors mentioned that the reason for this difference was the structures targeted in both applications. In another study, patients in the acute phase of HZ were divided into continuous epidural block and DRG PRF groups [21]. When the results were examined, more significant pain reduction was found in the DRG PRF group. The need for hospitalization due to the potential for significant hemodynamic changes, a higher risk of infection compared to DRG PRF, and the necessity to adjust epidural drug doses and monitor hemodynamics make epidural block less preferable than DRG PRF, especially in the elderly patient group. When evaluating the incidence of mild complications, the authors found a significantly lower complication incidence in the DRG PRF group. In our study, we applied DRG PRF to all patients. However, a notable point in this study is that, although the authors defined the acute phase as the first month from the onset, they designated the acute phase in the study design as the interval between 30 and 180 days from the onset. They did not make any comparisons in terms of duration. However, they reported that in both patient groups, although to a lesser extent in the DRG PRF group, there was a meaningful development of PHN. In our study, we grouped our patients based on the time elapsed since the onset of HZ-related pain, and we achieved the best results in the group where we applied DRG PRF in the first month. We did not observe the development of PHN in any of the patients in this group.

Our study was a retrospective analysis. Another limitation of our study is its small sample size. The other concern is that the procedures were performed by two different pain physicians. But their approach to the patient and their performing way of the procedure were the same.

## 5. Conclusion

We believe that interventional pain treatment should be applied as soon as possible in the elderly patient group who do not respond to first-line medical treatment or cannot tolerate medical treatment due to its side effects and who have a high risk of developing PHN compared to other age groups. We consider that DRG PRF, applied in the early period of medical treatment-resistant acute HZ, is safe and effective, preventing the progression to PHN.

## Figures and Tables

**Table 1:** The patients' basic clinical data.

	**Group 1**	**Group 2**
Age	73.79 ± 5.47	71.90 ± 5.27
Gender (female/male)	9/10	11/10
Time from pain onset (days)	22.16 ± 5.24	110.71 ± 43.33
Herpes zoster distribution (cervical/thoracic/lumbar)	4/12/3	4/14/3
Average pain scores (NRS)	7.37 ± 0.89	7.19 ± 0.81

Abbreviation: NRS, Numeric Rating Scale.

**Table 2:** NRS-11 score pre- and postoperation.

**NRS scores**	**Group 1**	**Group 2**	***p* value**
Baseline	7.37 ± 0.89	7.19 ± 0.81	0.516
1 week after	1.63 ± 0.89	3.67 ± 1.06	≤0.001
1 month after	1.68 ± 0.82	3.86 ± 1.15	≤0.001
3 months after	2 ± 0.81	4.1 ± 1.13	≤0.001
6 months after	2.32 ± 0.67	4.48 ± 1.36	≤0.001

## Data Availability

The data that support the findings of this study are available from the corresponding author (G.D) upon reasonable request.
